# Construction and Antiapoptosis Activities of Recombinant Adenoviral Expression Vector Carrying EBV Latent Membrane Protein 2A

**DOI:** 10.1155/2011/182832

**Published:** 2011-08-16

**Authors:** Xishuang Liu, Yu Gao, Bing Luo, Youan Zhao

**Affiliations:** ^1^Department of Gastroenterology, Qilu Hospital, Shandong University, Ji'nan, Shandong 250012, China; ^2^Department of Gastroenterology, The Affiliated Hospital of Medical College, Qingdao University, 16 Jiangsu Road, Qingdao, Shandong 266003, China; ^3^Department of Medical Microbiology, Medical College, Qingdao University, Qingdao, Shandong 266021, China

## Abstract

To evaluate the possible effects of LMP2A (EBV latent membrane protein 2A) on human gastric cancer cell line SGC-7901, LMP2A coding gene was subcloned into shuttle plasmid pAdTrackCMV to form transfer plasmid pAdTrackCMV-2A, which was linearized with PmeI and cotransformed into *E.coli* BJ5183 with adenovirus genomic plasmid of pAdeasy-1. The identified recombinant adenovirus plasmid DNA was digested with PacI and transfected into 293 cells to package recombinant adenovirus particles named vAd-2A. Then the expression and antiapoptosis activities of LMP2A on SGC-7901 infected with vAd-2A were analyzed. The vAd-2A was successfully constructed and identified by PCR, restriction digestion, and sequencing. LMP2A expression in SGC was identified by strong green fluorescence expression with fluorescence microscopic photograph and Southern blotting. The growth of LMP2A expressing SGC cells was apparently improved. Both cyclin E expression and S phase ratio in LMP2A expressing SGC cells were upregulated by cell cycle analysis and confocal microscopic analysis respectively. The replication-deficient recombinant adenovirus vector can express LMP2A antigen in SGC cells and inhibit their apoptosis. The results indicate that LMP2A might play an important role in pathogenesis of EBV-associated gastric cancer (EBVaGC). This study establishes a foundation for further study on EBVaGC and its gene therapy.

## 1. Introduction

Epstein-Barr virus (EBV), a gamma herpes virus, has been associated with a variety of human malignancies such as Burkitt's lymphoma (BL), nasopharyngeal carcinoma (NPC), and gastric cancer (GC) [1]. EBV is detected in the tissue of about 10% of gastric carcinoma cases throughout the world [2, 3]; furthermore, EBV is associated with 80%–100% of the rare lymphoepithelioma-like gastric carcinomas [4] and is also present in 35% of gastric stump carcinomas [5]. Latent membrane protein 2A (LMP2A) is an EBV-encoded protein that has been implicated in regulating viral latency and pathogenesis within infected cells [6]. Recent studies revealed that about 50% EBV-associated gastric carcinoma (EBVaGC) expressed LMP2A. Gastric cancer is one of the most common malignant tumors in the world. It is very difficult to remove and eliminate tumor cells completely by surgical, radio therapeutic, and chemotherapeutic methods. Therefore, developing an immunotherapeutic method to treat gastric cancer is of critical importance. LMP2A contains many potential T-cell activating epitopes which can trigger cytotoxic T lymphocytes (CTL), thus it is an ideal target gene of immunotherapy. However, the pathogenic role of LMP2A in tumor progression is not well understood. So making an intensive study of LMP2A is of important meaning to reveal the disease mechanism of EBV. In the present study, LMP2A gene was cloned into adenoviral vector to generate recombinant adenovirus, which could introduce LMP2A gene into gastric cancer cells, in order to evaluate the possible effects of LMP2A expression on human gastric cancer cell line. Our study provides more complete understanding of the role of LMP2A in EBV latency and tumorigenesis and establishes a foundation for further study on gastric cancer and its gene therapy.

## 2. Materials and Methods

### 2.1. Cell Culture and Medium

Type 1 EBV strain positive B95-8 cells were used for amplifying LMP2A gene, and human embryonic kidney cell line HEK293 were used for packing recombinant adenovirus. The human GC cell line SGC-7901 was purchased from ATCC. All cell lines were maintained in high glucose DMEM medium supplemented with 100 mL/L fetal bovine serum (FBS). The culture medium contained 100 U/mL of penicillin and 100 *μ*g/mL of streptomycin (GIBCO BRL, Gathersburg, Md) at 37°C in 5% CO_2_.

### 2.2. Target Gene and Primers

The RT-PCR product of LMP2A was obtained from B95-8 cell line genome RNA. The specific primers were as follows: 5′-GGCAGATCTATGGGGTCCCTAGAAATGGTG-3′ (sense strand) and 5′-GCGATATCTTATACAGTGTTGCGATATGG-3′ (antisense strand), and Bgl II and EcoR V restriction sites and protective bases were introduced into sense and antisense primers, respectively, as underlined. The predicted length of PCR product is about 1500 bp.

### 2.3. Recombinant Retroviral Vector Construction

PCR amplified target gene LMP2A and shuttle vector pAdTrack-CMV were digested with Bgl II and EcoR V. The digested products were purified and ligated with DNA ligation I to form transfer plasmid of pAdTrackCMV-2A. Then it was linearized by PmeI and cotransformed into BJ5183 bacterial cells with adenovirus genomic DNA plasmid of pAdeasy-1. The homologous recombinant adenoviral plasmid named pAd-2A was confirmed by PCR, restriction endonuclease analyses and sequencing. The control retroviral vector pAd-LacZ was constructed in the same manner.

### 2.4. Production and Propagation of Recombinant Adenoviruses in HEK293

Four micrograms of recombinant adenoviral vector DNA, after Pac I digestion and ethanol precipitation, were transfected into HEK293 cells in each 25 cm^2^ flask at 50%–70% confluence. Transfection was accomplished using the LipofectAMINE reagent from Life Technologies (Gaithersberg, Md). Transfected cells were monitored for GFP expression and collected 7–10 days after transfection, when about 1/3 cells appeared cytopathic effect (CPE), by scraping cells off flasks and pelleting them along with any floating cells in the culture. After centrifugation, the cell pellet was resuspended in 2.0 mL sterile phosphate-buffered saline (PBS) and lysed for four consecutive freeze/thaw cycles, and the virus was collected from the supernatant. One third of viral lysate was used to infect HEK293 cells in a 25 cm^2^ flask. The infection efficiency could be conveniently followed with green fluorescent protein (GFP). Three to four days later, viruses were harvested as described above. This process was repeated 1–3 times. At this point, viral titers were often high enough to use for gene transfer experiments in cultured cells. Viral titer was determined by virus titer determination kit following the manufacturers recommendations (Ben Yuan Zheng Yang Company, Beijing, China), which was up to 3.16 × 10^9^ pfu/mL.

### 2.5. Infection of SGC Cells with Recombinant Adenoviruses

When cultures of SGC cells were 70% confluence, the cells were incubated with recombinant adenoviruses at a proper MOI at 37°C overnight. The cells then were washed once with PBS, and fresh medium was added. At 4 days after infection, the infection efficiency was estimated by using fluorescence microscopy to detect GFP-expression. The infected SGC cells were harvested, and transcriptional expression of LMP2A in SGC cells were examined by RT-PCR followed by Southern blot analysis.

### 2.6. MTT

Cell viability was assayed by the incorporation of 3-(4, 5-dimethylthiazol-2-yl)-2, 5-diphenyl tetrasodium bromide (MTT) dye (Sigma). SGC cells were washed with PBS and seed into 96-well plates at 4 × 10^3^ cells/10 *μ*L per well. Used proper amount of virus that minimize cytotoxicity infect the cells after their adherence. Following 1h of serum starvation incubated with TGF-*β*1 (10 ng/mL), 100 *μ*L of MTT solution (5 mg/mL) was added to each well after 0, 24, 48, 72, 96, 120 h of incubation, respectively, and the plates were incubated for 4 h at 37°C. Then, 100 *μ*L DMSO was added to the wells, and the plates were incubated for 10 min at 37°C. The absorbance (A) was measured at 490 nm with a microplate reader (SPECTRA MAX 190; Molecular Devices).

### 2.7. RT-PCR and Southern Blot Analysis

Total RNA was extracted using Trizol RNA isolation reagent (Molecular Research Center, Cincinnati, Ohio). 1 *μ*g of total RNA was transcribed using the Reverse Transcription System (Promega, USA). Total RNA was treated with DNase I (Roche, Basel, Switzerland) to remove contaminating genomic and adenoviral DNA before cDNA was prepared. Three microliters of cDNA for each sample were used to perform the PCR. PCR on total RNA (before reverse transcription) was used to control for DNA contamination in samples. The sequences of primers were as follows: 5′-ATGACTCATCTCAACACATA-3′ (nt. 166874-166893), 5′-CATGTTAGGCAAATTGCAA-3′ (nt. 166380-166361). Product size was 280 bp. As probe, the oligonucleotide: ATCCAGTATGCCTGCCTGTA (nt. 166862-166881) was used. Southern blot hybridization was performed after RT-PCR for confirmation. The amplified products were electrophoresed in 2% agarose gel, transferred onto a Hybond N^+^ nylon membrane (Amersham Pharmacia Biotec, Ireland), and subjected to hybridization with 3′-end-DIG-labeled oligonucleotide probes. The hybridized signals were detected by alkaline phosphatase (AP) conjugated anti-DIG antibodies. The substrate of AP was CSPD (Roche Diagnostics, Germany). cDNAs from EBV immortalized lymphoblastoid cell lines (LCL) were used as positive controls, and those from EBV-negative SGC cells as negative controls.

### 2.8. Cell Cycle Analysis by Flow Cytometry

For flow cytometry analysis of the DNA content, SGC cells infected with vAd-2A, vAd-LacZ 24 h or 48 h after infection, or parental cells (more than 10^6^ cells) in either suspension or adhesion, were harvested and fixed in 70% ethanol for 3 h. The fixed cells were washed once with PBS and resuspended in PBS containing 10 *μ*g/mL DNase-free RNase and 25 *μ*g/mL propidium iodide. DNA fluorescence was measured using a Coulter EPICS Profile II flow cytometer equipped with an argon laser to give 488 nm light. Data from 10^4^ cells were collected, and the percentages of cells in the S phase of the cell cycle were determined by Multicycle software (Phoenix Flow Systems). Analysis was repeated three times for each cell group.

### 2.9. Confocal Laser Scanning Microscopy

For confocal microscopic analysis, cover slips containing indicated adenovirus infected SGC cells were washed twice with PBS and fixed with paraformaldehyde (4% in PBS) for 30 min. Fixation was stopped by incubation in glycine (0.1 M in PBS) for 10 min, and the cells were permeabilized with Triton X-100 (0.3% in PBS) for 30 min. Free binding sites were saturated with 1% bovine serum albumin solution for 1 h at room temperature and then incubated with primary antibody (rabbit monoclonal anticycinE, diluted 1 : 200 in PBS). After incubation for 1 h at 37°C, the cells were washed 3 times with PBS and then incubated for 1h at 37°C with the secondary antibody (Rhodamine-conjugated Affinipure Goat AntiMouse IgG, 1 : 200 dilutions). Immunofluorescence and direct fluorescence (EGFP) images were taken using a confocal microscope (Zeiss).

### 2.10. Statistics Analysis

All data are expressed as means ± SD. Statistical comparisons were made using the analysis of variance. *P* < 0.05 was considered significant.

## 3. Results

### 3.1. Analysis of Recombinant Plasmid pAd-2A

**Figure 1:**
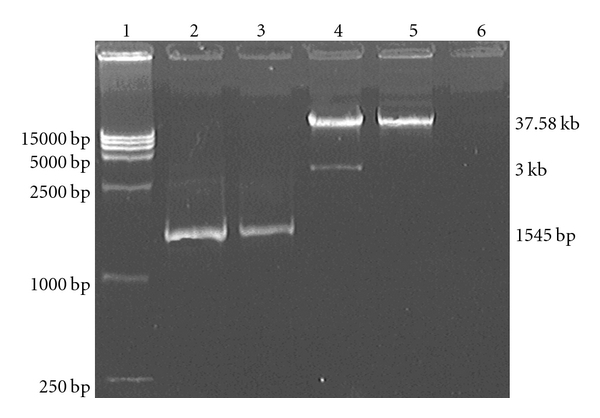
Restrictive analysis of pAd-2A with Pac I and PCR product of target gene. lane 1: Marker DL15000; lane 2: PCR product of positive control (EBV positive B95-8 cell); lane 3: PCR product of pAd-2A; lane 4: pAd-2A/Pac I; lane 5: pAd-2A; lane 6: PCR product of pAd-GFP.

The fragments of PCR amplification and Pac I digestion of plasmid pAd-2A digested were 1500 bp, 37.58 kb, and 3.0 kb, respectively (Figure 1). All the products were detected by 1% agarose gel electrophoresis as predicted. The expression of the LMP2A gene was verified by gene sequencing.

### 3.2. Preparation of Recombinant Adenovirus

**Figure 2:**
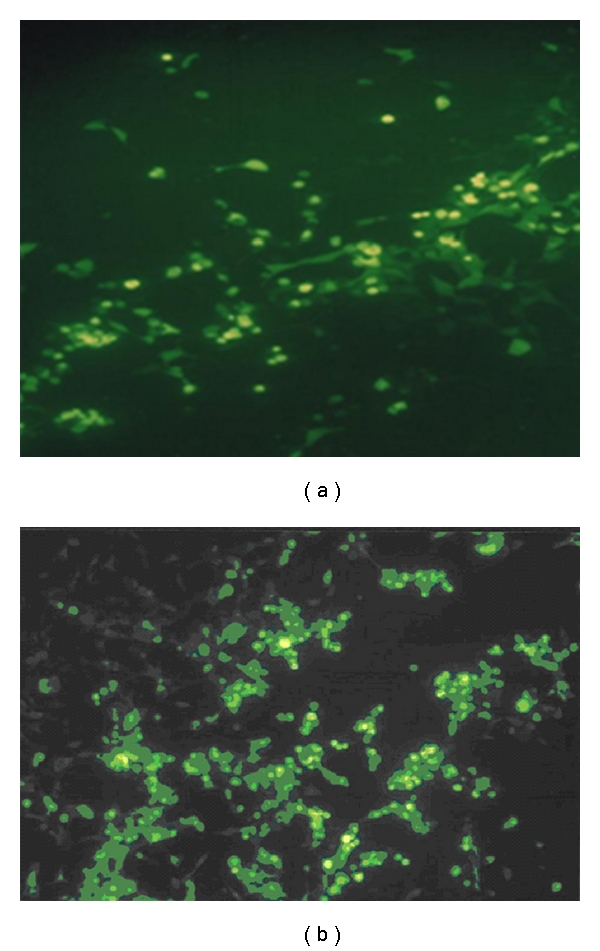
Adenovirus-producing foci after transfection of 293 cells, (a) 2 days after transfection; (b) 7 days after transfection (CPE).

**Figure 3:**
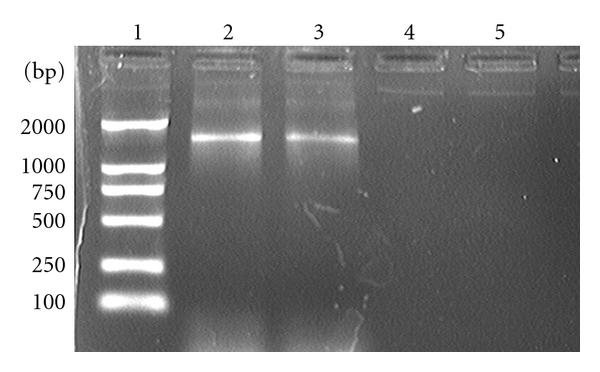
Transcriptional expression of LMP2A in pAd-2A transfected 293 cells. Lane 1: Marker DL2000; lane 2: positive control (EBV-positive LCL); lane 3: pAd-2A transfected; lane 4: pAd-LacZ transfected; lane 5: negative control (PCR on total RNA without reverse transcription).

Expression was visible 24 h after transfection in 20%–30% of the cells, representing the fraction of the population that was transfected. Comet-like foci, visualized with GFP fluorescence, began to appear at 4-5 days after transfection, indicating adenovirus was produced successfully (Figure 2). The adenovirus-mediated gene LMP2A expression was verified in HEK 293 by RT-PCR. The expected 1500-bp fragment was amplified from pAd-2A transfected SGC cells, but not from pAd-LacZ transfected cells (Figure 3).

### 3.3. Transcriptional Expression of LMP2A in SGC Cells

**Figure 4:**
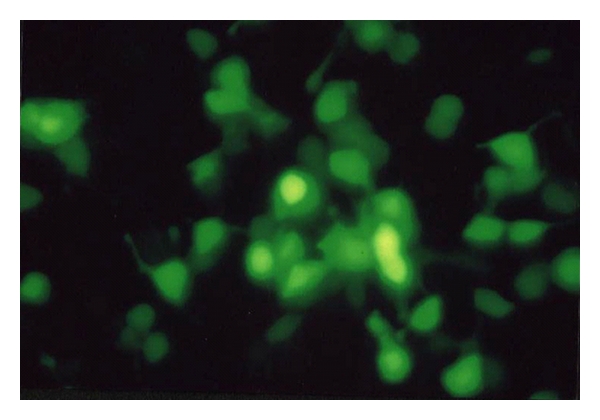
GFP expression in target cells infected with vAd-2A.

**Figure 5:**
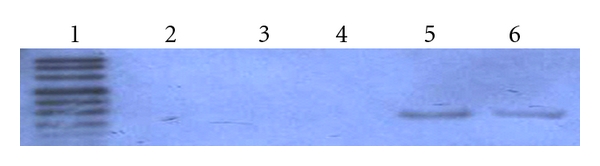
Detection of LMP2A expression in SGC cells by PCR-Southern blotting. Lane 1: DIG-labeled DNA molecular weight marker VIII (Roche); lane2: SGC cells; lane 3: vAd infected SGC cells; lane 4: negative control (PCR on total RNA without reverse transcription); lane 5: vAd-2A-infected SGC cells; lane 6: positive control (EBV-positive LCL).

**Figure 6:**
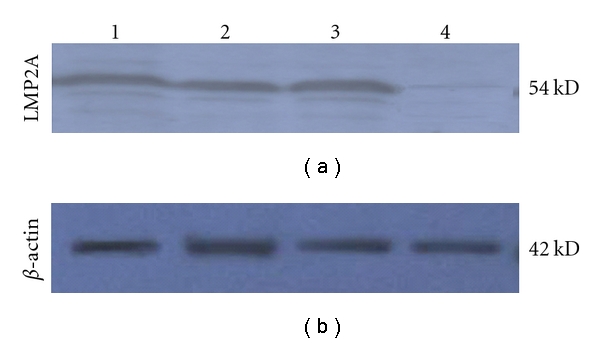
The expression of LMP2A protein in vAd-2A-infected SGC cells by Western Blotting *β*-actin was used for internal control, lane 1, 2, 3: vAd-2A-infected SGC cells; lane 4: vector control (SGC-GFP).

GFP expression was visualized by fluorescence microscopy 48 h after transfection, and the infection rate can reach up to 90% 4 days postinfection, indicating vAd-2A could effectively infect SGC cells (Figure 4). Total RNA was extracted from the test and control cells, and LMP2A-specific primers were used for amplification by RT-PCR. PCR products were hybridized by Southern blotting analysis with a LMP2A-specific probe. The expected 280-bp fragment was amplified from vAd-2A-infected SGC cells, but not from parental and vector control SGC cells (Figure 5). The expression of LMP2A protein in vAd-2A-infected SGC cells was also verified by Western blotting (Figure 6).

### 3.4. MTT

The cell growth was analyzed by MTT assay. The viable cell number of vAd-2A-infected SGC cells increased significantly (*P* < 0.05) compared with SGC cells at the same time point, the antiapoptosis ratio in 0 h, 24 h, 48 h, 72 h, 96 h, and 120 h is −1.105%, 23.944%, 21.513%, 20.826%, 24.874%, and 16.029%, respectively, as shown in Table 1. vAd infected SGC cells had no significant change.

### 3.5. LMP2A Increased the Level of Cyclin E

**Figure 7:**
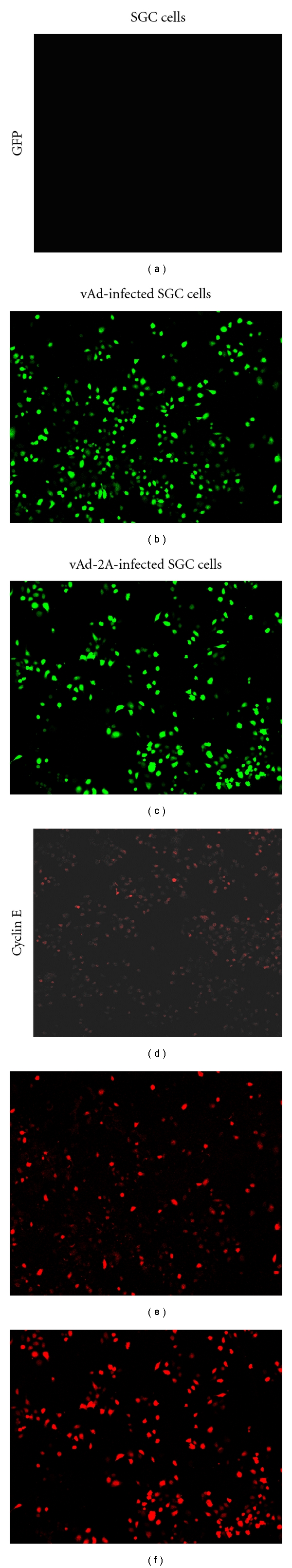
Confocal analysis for cyclin E on SGC cells.

The infection rates of vAd and vAd-2A-infected SGC cells were both about 80% after 48 h infection visualized with GFP fluorescence (b, c) while no GFP was visualized in SGC cells (a), as shown in Figure 7. The level of cyclin E in vAd-2A-infected SGC cells increased significantly, and almost all of the infected cells could express cyclin E (f). These results suggested that LMP2A could enhance the expression of cyclin E in SGC cells significantly.

### 3.6. The LMP2A-Positive SGC Cells Have an Increased Number of Cells in S Phase

The percentage of LMP2A-positive and LMP2A-negative cells in S phase of the cell cycle is shown in Table 2. The data showed a significant increase in the percentage of G1-phase-arrested cells in SGC cells after transduction with an adenovirus carrying LMP2A gene in comparison with parental and vector control SGC cells (*P* < 0.01).

## 4. Discussion

Gastric cancer, with clinical prognosis, is the second leading cause of cancer-related mortality worldwide [7]. Recently, great attention has been paid to the association of EBV infection and gastric carcinoma. EBV is detected in the tissue of about 10% of gastric carcinoma cases throughout the world. In each case, 100% of carcinoma cells are infected with EBV. Analysis of EBV in carcinoma biopsies indicates that carcinoma is formed by the proliferation of a single EBV-infected cell [2]. These findings suggest that EBV plays an important role in the development of EBV-positive gastric carcinomas. LMP2A gene is expressed in vivo in humans with latent infections and most EBV-related malignancies [8–11]. Recent studies revealed that about 50% EBV-associated gastric carcinoma (EBVaGC) expressed LMP2A. Due to this persistent expression, LMP2A is thought to play an important role in EBV latency and may be an important risk factor in EBV-associated diseases.

It has been reported that LMP2A is involved in blocking B-cell specific signaling pathways and calcium mobilization, which might be advantageous for maintaining latent patterns of EBV infection and inhibiting EBV reactivation [12]. LMP2A is also reported to transform epithelial cells, greatly affect cell growth and differentiation pathways in a human keratinocyte cell line (HaCaT), and inhibit transforming growth factor-*β*1-induced apoptosis in HSC-39, through activation of the phosphatidylinositol-3-kinase (PI3K)-Akt pathway [13, 14]. EBV latent membrane protein 2A activates beta-catenin signaling in epithelial cells [15]. The emerging importance role of LMP2A in both B-cell and epithelial cell growth led us to evaluate the effect of LMP2A on cell cycle regulation in a GC cell line. TGF-*β* is a multifunctional cytokine that plays important roles in regulating cell growth and differentiation in many biological systems [16]. TGF-*β* can induce immunoglobulin A class switching and play an important role in differentiation, growth, matrix formation, and the regulation of immune and inflammatory responses [17, 18]. It is an inducer of apoptosis in BL and some gastric carcinoma cell lines [19–22]. Makoto Fukuda et al. demonstrated that LMP2A effectively suppressed TGF-*β*1-induced apoptotic death of B cells and gastric carcinoma cells, and the antiapoptosis effect was not mediated through decreased expression of TGF-*β* receptors. In our study, the data of MTT showed the viable cell number of vAd-2A-infected SGC cells increased significantly, compared with parental SGC cells at the same time point. The antiapoptosis ratio of vAd-2A-infected SGC cells in 0 h, 24 h, 48 h, 72 h, 96 h and 120 h is −1.105%, 23.944%, 21.513%, 20.826%, 24.874% and 16.029%, respectively. In accordance with Makoto Fukuda et al., this result proved the antiapoptosis activities of LMP2A on SGC cells. 

Abnormalities in cell cycle regulation lead to uncontrolled cell proliferation and the transformation into cancer cells [23, 24]. Cell cycle analysis indicated that vAd-2A-infected SGC cells had significantly more cells in S phase in comparison with parental and vector control SGC cells. Cyclins, CDKs, and CDKIs play important roles in controlling major checkpoints in the mammalian cell cycle [25]. Cyclin E overexpression occurred in 50%–60% of GC and adenocarcinomas [26] and is essential for progression from early to late G1 [27]. The cyclin E expressions of SGC detected by confocal microscopic analysis showed a positive linear correlation with S phase. Liang Bin et al. analyzed the relationship between cyclin E/CDK2 expression and clinicopathological characteristics of the tumors, indicating that overexpression of cyclin E protein was correlated with poorer differentiation advanced tumor stage and lymph node involvement [28], which was consistent with the report of Sakagnchi et a1. [29].

In summary, these results suggest that upregulated expression of cyclin E and S phases caused by LMP2A may be associated with the malignant potential in gastric cancer as well as potentiate metastases. LMP2A could increase GC cell proliferation via upregulating the expression of cyclin E and CDK2 which induced S phase arrest and inhibit apoptosis of SGC cells. These studies have provided a more complete understanding of the role of LMP2A in tumorigenesis of GC and established a foundation for further study on gastric cancer and its gene therapy.

## Figures and Tables

**Table 1:** Antiapoptosis activity of LMP2A on SGC cells.

Cell lines	A (means ± SD) and antiapoptosis ratio (%)					
	0 h	24 h	48 h	72 h	96 h	120 h
SGC cells-LMP2A	0.179 ± 0.020	0.352 ± 0.006	0.51 ± 0.015	0.731 ± 0.014	0.743 ± 0.007	0.63 ± 0.022
	−1.105%*	23.944%	21.513%	20.826%	24.874%	16.029%
SGC cells-vect	0.175 ± 0.017	0.290 ± 0.023	0.42 ± 0.019	0.588 ± 0.016	0.577 ± 0.018	0.52 ± 0.013
	−3.315%	2.112%	0.946%	−2.810%	−3.025%	−3.643%
SGC cells	0.181 ± 0.032	0.284 ± 0.012	0.42 ± 0.013	0.605 ± 0.028	0.595 ± 0.021	0.54 ± 0.027
	100%	100%	100%	100%	100%	100%

**Table 2:** Influence of LMP2A expression on cell cycle (FCM analysis) (means ± Std).

Cell lines	S phase (%)	
	24 h	48 h
SGC cells	13.227 ± 4.552	12.53 ± 3.206
SGC cells-LMP2A	14.035 ± 3.371	12.891 ± 2.613
SGC cells-vect	35.152 ± 5.109*	25.593 ± 4.056**

*F = 23.9048, *P = *0.0014 < 0.01, **F = 14.8453, *P = *0.0048 < 0.01, SGC-LMP2A versus SGC or SGC-vect.
